# The Round Vacuum Chamber

**Published:** 1903-11

**Authors:** P. B. M’Cullough

**Affiliations:** Philadelphia


### THE ROUND VACUUM CHAMBER.

BY P. B. M'CULLOUGH, D.D.S., PHILADELPHIA.

If the vacuum chamber were placed in a plate on the right side midway between the median line and the ridge, the resistance to force upon the teeth would be greatest on the left side, as the power of a lever is multiplied by its length.

A painter's scaffold may be suspended by a rope caught over the end of a board extending beyond the edge of a roof a few inches, insurance against the scaffold falling being one with the length of the board resting upon the roof. Therefore the farther the chamber is from the ridge the more secure the plate against tilting.

As the force applied in the molar region will be the same on either side, the middle of the chamber must be over the median line of the vault. In order that the greatest possible surface of perfect adaptation of the plate may be attained, the chamber must be the smallest possible consistent with the exhaust of a certain volume of air.

And in order that the chamber may be farthest away from the line of the application of force, and that the greatest field of plate contact may be attained between the edge of the chamber and the ridge, it must cover as small an area as possible.

. With the form of chamber having a flat side parallel with the back edge of the plate, the ingress of air is easiest and the relief of the vacuum quickest because the edge of the chamber throughout its entire width offers an extended surface for the ingress of air in the direction from which the vacuum is most naturally relieved. With the round chamber but one point is nearest the edge of the plate, hence the dam is most complete.

The leverage on a plate is greatest in the incisal region, and as the support against tilting would be in proportion as the chamber is back, it should be so placed, modified by the provision that its nearest point to the back edge of the plate should not be less than one-fourth to three-eighths of an inch; and further, that the tissues are more sensitive in the vault than anteriorly at the rugæ where the power of suction is greatest.

The diameter of the round vacuum chamber patterns should be five-eighths, three-fourths, and seven-eighths of an inch, and one-sixteenth of an inch in thickness. The pattern may be adapted to the model by first concaving it with the fingers free handed, then upon the model with two burnishers.

It can be secured in place by forcing the three-eighths-inch point end of two pins through each side of the metal on a line with the centre, grasping one at a time with pliers, and directing each point in an opposite direction, slanting to one side, using counterforce with a finger. Then burnish over the perforation.

